# Gastrodin ameliorates atherosclerosis by inhibiting foam cells formation and inflammation through down-regulating NF-κB pathway

**DOI:** 10.1186/s12986-022-00722-z

**Published:** 2023-02-09

**Authors:** Xiaofei Xue, Fulei Li, Mengke Xu, Bowen Chen, Yanyan Zhao, Mengyu Wang, Ling Li

**Affiliations:** 1 Department of Cardiology, The First Affiliated Hospital of Zhengzhou University, Zhengzhou, Henan China; 2 Department of Infectious Diseases, The First Affiliated Hospital of Zhengzhou University, Zhengzhou, China

**Keywords:** Atherosclerosis, Macrophage, Foam cell formation, Inflammation, Gastrodin

## Abstract

**Background:**

Gastrodin is an effective polyphenol extracted from Chinese natural herbal *Gastrodiae elata Blume*, which exhibits antioxidant and anti-inflammatory effects. It has been reported to benefit neurodegenerative diseases, but the effect of Gastrodin on atherosclerosis and the underlying mechanisms remain elusive. The aim of this study is to investigate the function and mechanism of Gastrodin in atherosclerosis.

**Methods:**

Atherosclerosis mouse model was established by fed low density lipoprotein receptor-deficient (*Ldlr*^*−/−*^) mice with a high fat diet (HFD, 20% fat and 0.5 cholesterol) for 8 weeks and Gastrodin was administered daily via oral gavage. Plasma lipid levels were measured using commercial kits. *En face* and aortic sinus lipid accumulation were analyzed with Oil Red O staining. In vitro cell models using foam cell formation model and classical atherosclerosis inflammation model, macrophages were incubated with oxygenized low-density lipoproteins (ox-LDL) or lipopolysaccharide (LPS) in the presence of different concentration of Gastrodin or vehicle solution. Foam cell formation and cellular lipid content were evaluated by Oil Red O staining and intracellular lipids extraction analysis. Gene expression and proteins related to cholesterol influx and efflux were examined by quantitative reverse transcription PCR (RT-qPCR) and western blotting analysis. Furthermore, the effect of Gastrodin on LPS induced macrophage inflammatory responses and NF-κB pathway were evaluated by RT-qPCR and western blotting analysis.

**Results:**

Gastrodin administration reduced the body weight, plasma lipid levels in *Ldlr*^*−/−*^ mice after fed a high fat diet. Oil Red O staining showed Gastrodin-treated mice displayed less atherosclerosis lesion area. Furthermore, Gastrodin treatment significantly ameliorated ox-LDL-induced macrophage-derived foam cells formation through suppressing genes expression related to cholesterol efflux including scavenger receptor class B and ATP-binding cassette transporter A1. Moreover, Gastrodin markedly suppressed pro-inflammatory cytokines secretion and LPS induced inflammatory response in macrophage through downregulating NF-κB pathway.

**Conclusions:**

Our study demonstrated that Gastrodin attenuates atherosclerosis by suppressing foam cells formation and LPS-induced inflammatory response and represents a novel therapeutic target for the treatment of atherosclerosis.

## Background

Atherosclerosis, which the major cause of cardiovascular disease (CVD) and stroke, has threatened people’s life [1]. The progression of atherosclerosis involves hyperlipidemia, hyperglycemia, inflammation and reactive oxygen species [2–4]. Numerous studies on its pathogenesis have illustrated that atherosclerosis is an inflammatory disease of the blood vessels mediated by endothelial injury, inflammatory infiltration and foam cell formation [5–7]. The initial step for atherosclerosis is endothelial cells injury and dysfunction. And then macrophage-derived foam cells formation and pro-inflammatory cytokine secretion play a crucial role in atherosclerosis progression [8–10].

Gastrodin is an effective polyphenol extracted from Chinese natural herbal *Gastrodiae elata Blume*, which exhibits antioxidant and anti-inflammatory effects [11]. Gastrodin has been used to treat neurodegenerative diseases in clinical practice, such as epilepsy, migraine and convulsions [12–14], the underlying mechanisms are related to apoptosis, oxidative stress and neurotransmitter release. In addition, Gastrodin has been shown to lower blood pressure and alleviate cardiac hypertrophy [15, 16]. In the context of cardiac hypertrophy, Gastrodin was reported to target insulin-like growth factor type 2 gene (IGF2) and its receptor IGF2R [15]. Additionally, Gastrodin has been demonstrated to reduce plasma lipids levels and ameliorate nonalcoholic fatty liver disease (NAFLD) through activating the AMPK signaling pathway [17]. Recent study indicated that Gastrodin affect the foam cell formation through regulation of lysosomal biogenesis and autophagy in vitro cell model [18]. However, the effect of Gastrodin on atherosclerosis in vivo and the underlying mechanism remains unknown.

In the present study, we investigate the anti-atherosclerosis effect of Gastrodin in vivo, using *Ldlr*^*−/−*^ mice fed a high fat diet (HFD). Furthermore, in vitro experiments, Gastrodin ameliorated ox-LDL induced foam cell formation and LPS-induced inflammation response. Mechanistically, on one hand, Gastrodin treatment represses SRA-mediated lipid influx in macrophages, but augments gene expression related to cholesterol efflux including SRB-I and ABCA1. On the other hand, Gastrodin inhibits LPS-induced inflammation response through downregulating NF-κB signaling pathway.

## Methods

### Materials

Gastrodin (purity > 98%) was purchased from Sigma (St. Louis, MO, USA). Antibodies against GAPDH (Cat. No. ab-9485), Histone H1 (Cat. No. ab-4270), TNF-α (Cat. No.ab-381238)), NF-κB p65 (Cat. No.ab-32536)), SR-B1 (Cat. No. ab-106572), ABCA1 (Cat. No.ab-18180) and CD36 (Cat. No. ab-252923) were purchased form Abcam CO (Cambridge, USA). IL-1β (Cat. No. 12242) were purchased form Affinity Biosciences (OH, USA). IL-18 (Cat. No. A16741) were purchased from ABclonal Technology (Wuhan, China). The secondary antibodies HRP-conjugated anti-mouse lgG and HRP-conjugated anti-rabbit lgG were purchased from Affinity Biosciences (OH, USA). *Animals* Eight-week-old male *Ldlr*^*−/−*^mice were purchased from Cyagen Laboratories. The mice were fed with a high fat diet (HFD, 20% fat and 0.5% cholesterol). Gastrodin-treated group was administered with Gastrodin daily via oral gavage at doses of 50 mg/kg, and normal saline for control group for 8 weeks. Mice were weighed once every week, and food intake was monitored throughout the experiments. All animals were maintained on a 12:12 h light-dark cycle and have free access to water and food.

All experiments involving mice were approved by the Institutional Animal Care Research Advisory Committee of the National Institute of Biological Science (NIBS) and Animal Care Committee of Zhengzhou University.

### Plasma lipid measurements

Blood was obtained by retro-orbital bleeding after overnight fasting. Plasma triglyceride (TG) and total cholesterol (TC) were measured by enzymatic methods according to the manufacturer’s instructions (Sigma kits, USA).

### Atherosclerosis lesion analysis

Hearts and proximal aortas were obtained and fixed in 4% paraformaldehyde. And aortas were removed from the iliac artery bifurcation to the origin at the heart. *En face* lipid accumulation were stained with Oil Red O and atherosclerotic lesions were analyzed. The upper sections of hearts were embedded in OCT medium. The aortic sinus Sects. (4 μm) were prepared and stained with Oil Red O. The lesion of aortas and aortic sinus were analyzed by using Image J software.

### Immunohistochemistry

Macrophage contents in atherosclerotic lesion were measured using immunohistochemistry staining. Briefly, aortic sinus sections were incubated with 3% H_2_O_2_ for 10 min and blocked with 3% BSA (Sigma) for 1 h and incubated with anti-F4/80 antibody (1:200, Abcam, Inc., CA, USA; Cat. No.ab-300421) overnight at 4 °C. After incubating with anti-rabbit lgG for 1 h at room temperature, slides were developed with 3,3′-diaminobenzidine (DAB Quanto Kit, TA-060-QHDX, ThermoFisher) and stained with heamatoxylin. Images were recorded using a light microscope.

### Measurement of inflammatory cytokines by ELISA

Aorta was freshly isolated and homogenized in Tris buffer. And then aorta homogenate was centrifuged at 12,000 × g for 5 min. The supernatant was analyzed for protein concentration. The inflammatory cytokines in aorta homogenate were measured by enzyme-linked immunosorbent assay (ELISA) (R&D Systems, Abingdon, UK) in accordance with the manufacturer’s instructions.

### Cell culture

C57BL/6 mice were intraperitoneally injected with 4% solution of thioglycollate media. Three days after injection, peritoneal macrophages were isolated and cultured in RPIM 1640 with 10% fetal bovine serum with a humidified atmosphere of 5% CO_2_ at 37 °C.

### MTT cell viability assay

Cell metabolic activity was analyzed by the MTT reduction assay as per manufacturer’s protocol after treated with different concentrations of gastrodin for 24 h. Briefly, cells at a density of 4 × 10^4^ cells per well were cultured in 96-well plates for 24 h. Cells were incubated with MTT solution for 4 h at 37 °C. DMSO was used to dissolve the insoluble formazan product. The absorbance values at 570 nm were then read using a microplate reader (Bio-Rad, Hercules, CA, USA). All experiments were repeated at least three times.

### Foam cell formation

Macrophages were cultured on chamber slides in 12-well palates. Cells were incubated with 20 μg/ml ox-LDL (Yiyuan Biotech) for 24 h to induce the formation of foam cells.

*Intracellular cholesterol measurement* The peritoneal macrophage cultured in 12-well palates was incubated with 20 μg/ml ox-LDL for 24 h. Then cells were washed 3 times with PBS, and then cholesterol and triglyceride were determined using commercial kits from Applygen Technologies (Beijing, China).

### Western blotting

Total protein and nuclear protein were extracted from cells and murine tissues. The protein concentration was detected by using a BCA protein assay kit. Equal amounts of protein (25 μg) were separated on SDS-PAGE gel and electro-transferred onto a polyvinylidene difluoride membrane (PVDF). Next, PVDF membranes were blocked with 5% fat-free milk for 1 h, and then incubated with primary antibodies overnight at 4 °C. After incubating with secondary antibodies at room temperature, the optical density of the bands was visualized by an ECL system (Pierce). Data was normalized to GAPDH or Histone H1 levels.

### Nuclear protein isolation

Cells were washed with PBS and lysed by cell lysis buffer (10 mM Tris, 10 mM KCl, 1.5 mM MgCl_2,_ and 1 mM DTT in 1 × complete protease inhibitor cocktail), and nuclei ware enriched by centrifugation at 4700 g for 10 min at 4 °C. Then, the nuclear proteins were extracted with chromatin digestion buffer (20 mM Tris (pH 7.5), 15 mM NaCl, 60 mM KCl, 1 mM CaCl2, 5 mM MgCls, 30 mM sucrose and 0.4% NP40) after incubating at 4 °C for 30 min. A BCA kit was used to quantify the unclear protein concentration.

### RNA isolation and mRNA expression using quantitative reverse transcription PCR (RT-qPCR)

Total RNA from the cells was extracted using Trizol reagent (cat. No. 15596026; Invitrogen; Thermo Fisher Scientific, Inc.), as per the manufacturer’s protocol. First strand cDNA was generated by using an RT kit (Invitrogen; Thermo Fisher Scientific, Inc.). qPCR was then performed using an opticon continuous fluorescence detection system with SYBR Green fluorescence (Molecular Probes, Eugene, USA). The RT-qPCR thermocycling parameters were as follows: initial denaturation at 94 °C for 5 min, followed by 40 cycles for 5 s at 94 °C, for 30 s at 60 °C, and for 30 s at 72 °C, and a final extension of 30 s at 72 °C. A single melting curve peak confirmed the presence of a single product. GAPDH was used as the reference control gene. Results were expressed as fold differences relative to GAPDH using the 2-ΔΔCq method. All the primers were synthesized by Sangon Biotech (Shanghai, China) and the sequence are listed in Table 1.

**Table 1:** Primer list for quantitative real-time PCR

Gene name	Forward primer (5′-3′)	Reverse primer (5′-3′)
F4/80	TTTCCTCGCCTGCTTCTTC	CCCCGTCTGTATTCAACC
TNFα	CTGTGAAGGGAATGAATGTT	CAGGGAAGAATCTGGAAAGGTC
MCP1	TCCCAATGAGTAGGCTGGA	AAGTGCTTGAGGTGGTTGT
IL-1β	AGGCTCCGAGATGAACAA	AAGGCATTAGAAACAGTCC
TGF-β1	GGCGGTGCTCGCTTTGTA	TCCCGAATGTCTGACGTAT
IL-6	TAGTCCTTCCTACCCCAATTTCC	TTGGTCCTTAGCCACTCCTTC
IL-18	GACTCTTGCGTCAACTTCAAGG	CAGGCTGTCTTTTGTCAACGA
SRA	GAACAACATCACCAACGACC	GACCAGTTTGTCCAGTAAGCC
SRB1	GCCTCTGTTTCTCTCCCACC	CTGTCCGCTGAGAGAGTCCT
CD36	CTTGAAGAAGGAACCACTGCT	CGAACTCTGTATGTGTAAGGACCT
ABCA1	CATCCTACAGTGCTTCCTCATTAG	CGAAATACTCACAGCCGAATC
ABCG1	TGCCTCACCTCACTGTTCA	TCTTTGACCATCTCTCGTCTG
NF-κB	ATGGCAGACGATGATCCCTAC	TGTTGACAGTGGTATTTCTGGTG
GAPDH	TCCTTGGAGGCCATGTGGGCCAT	TGATGACATCAAGAAGGTGGTGAAG

### Statistical analysis

The data are presented as means ± standard error of mean (SEM). SPSS 21.0 was used to perform statistical analysis of the data. Statistical differences were calculated with the 2-tailed Student t test when comparing 2 conditions, and ANOVA was used when comparing > 2 conditions. A value of P < 0.05 was considered statistically significant.

## Results

### Gastrodin ameliorated plasma lipid levels in Ldlr^***−/−***^ mice fed a HFD diet

**Fig. 1:**
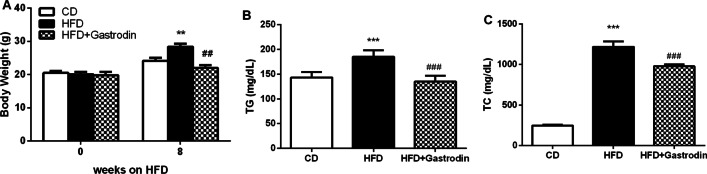
Effect of Gastrodin on body weight and plasma lipid levels of *Ldlr*^*−/−*^ mice fed with HFD. Body weight (**A**), plasma TG (**B**) and TC (**C**) levels. Data are presented as mean ± SEM, n = 8, ^**^P < 0.01, ^***^P < 0.001 for HFD mice versus CD mice. ^##^P < 0.01, ^###^P < 0.001 for HFD mice versus HFD + Gastrodin mice

*Ldlr*^*−/−*^ mice were treated with HFD diet for 8 weeks and body weight were monitored. Our data showed that HFD group mice gained more weight than control mice and Gastrodin administration significantly reversed increase in body weight in HFD group mice (Fig. 1A). Then we analyzed plasma lipid levels. As shown in Fig. 1B and C, HFD induced hyperlipidemia, and Gastrodin treatment significantly lower the levels of TG and TC. These data demonstrated that Gastrodin administration can reduce body weight and plasma lipid level.

### Gastrodin alleviates atherosclerosis lesion and macrophage accumulation in Ldlr^***−/−***^ mice

**Fig. 2:**
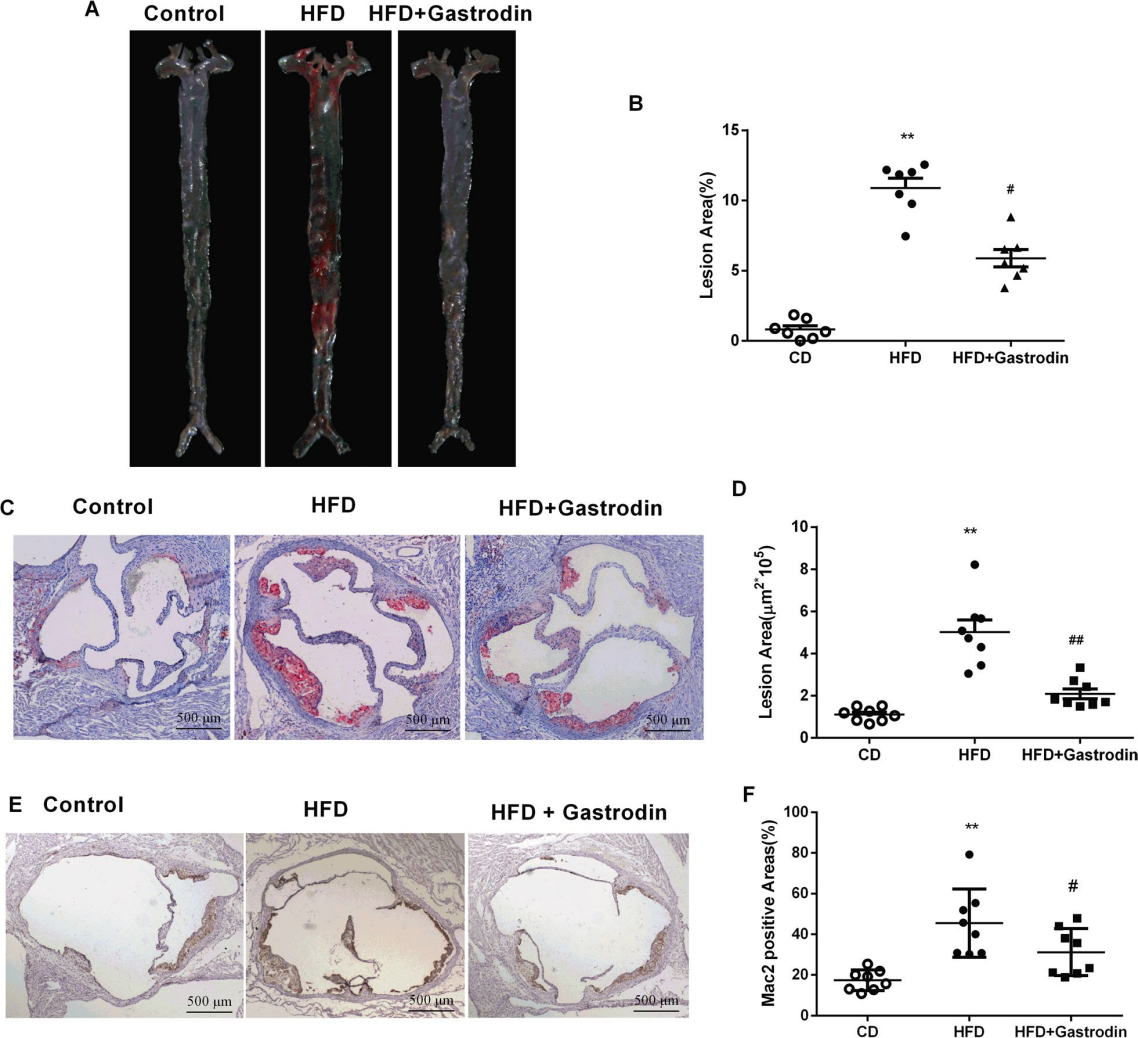
Gastrodin alleviates atherosclerosis in HFD fed *Ldlr*^*−/−*^ mice. **A** and **B** Representative *en face* images of Oil red O-stained aorta and quantification of lesion area. **C** and **D** Representative aortic root sections stained with Oil red O and quantification of aortic lesion areas. **E** and **F** Representative F4/80 immunostaining and quantification of aortic sinus lessions. Data are presented as mean ± SEM, n = 8, ^*^P < 0.05, ^**^P < 0.01 for HFD mice versus CD mice. ^#^P < 0.05, ^##^P < 0.01 for HFD mice versus HFD + Gastrodin mice

Atherosclerosis was induced in *Ldlr*^*−/−*^ mice fed a HFD for 8 weeks and then the therapeutic effect of Gastrodin on atherosclerosis was evaluated. As shown in Fig. 2A and B, *en face* analysis and quantification of the aorta lesions indicated that HFD induced obvious atherosclerosis lesions in aortic arch branches and abdominal aorta, and intervention with Gastrodin halted atherosclerosis lesion progression. Quantification of Oil red O staining of aortic roots sections showed reduced plaque size in Gastrodin group mice compared to HFD group mice (Fig. 2C and D). These results demonstrated that Gastrodin might have a protective role in atherosclerosis progression. Formation of macrophage-derived foam cells is one hallmarks of the initial stages of atherosclerosis. Then, we evaluate the effect of Gastrodin on macrophage accumulation in atherosclerosis lesions. By performing immunohistochemical staining of aorta sinus sections with macrophage antibody F4/80, the positive areas were analyzed with Image J. As shown in Fig. 2E, there were more F4/80 positive cells in the atherosclerosis lesions of HFD group mice when compared with control group mice, and Gastrodin treatment significantly alleviated macrophage accumulation in atherosclerosis lesion sizes (Fig. 2E and F). These findings demonstrated that Gastrodin not only attenuates atherosclerosis lesions but also reduces foam cell formation.

### Gastrodin reduced inflammatory cytokines in Ldlr^***−/−***^ mice

**Fig. 3:**
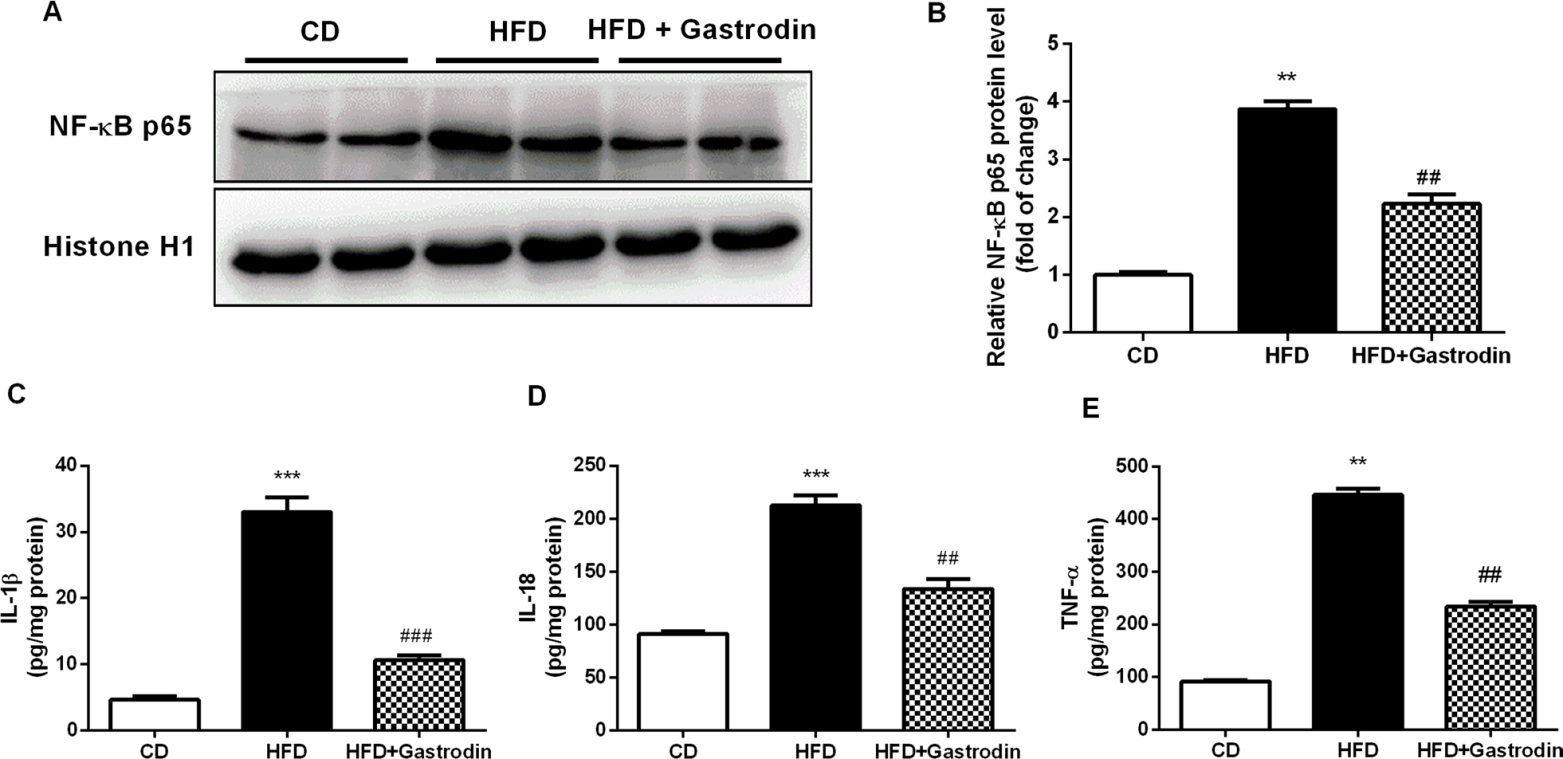
Gastrodin attenuates inflammatory cytokiines in HFD fed *Ldlr*^*−/−*^ mice. **A** and **B** Protein level and quantitative analysis of NF-κB p65 in the homogenate of aortic arch. C, D and E. Plasma IL-1β, IL-18 and TNF-α levels were measured by the ELISA assay. Data are presented as the mean ± SEM, ^**^P < 0.01, ^***^P < 0.001 for HFD mice versus CD mice. ^##^P < 0.01, ^###^P < 0.001 for HFD mice versus HFD + Gastrodin mice

Considering the important role inflammation plays in atherosclerosis. We then analyzed the level of IL-1β, IL-18, TNF-α and NF-κB p65 in arteries of atherosclerosis. As shown in Fig. 3A and B, we found that Gastrodin decreased the expression levels of aorta TNF-α and NF-κB p65, leading to reduced inflammatory cytokines levels including IL-1β, IL-18 and TNF-α (Fig. 3C, D and E).

### Effect of Gastrodin on cell viability

**Fig. 4:**
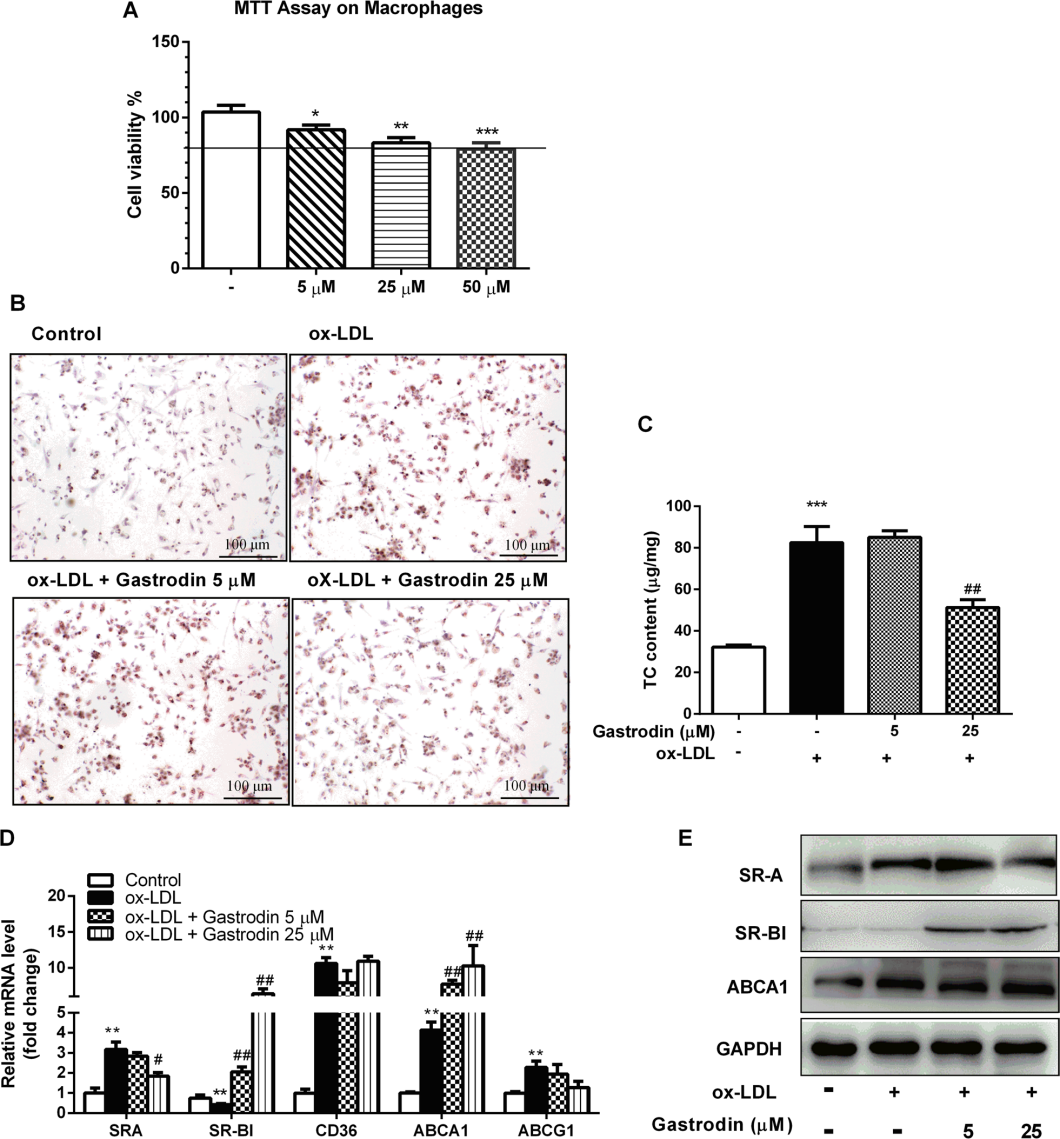
Effect of Gastrodin on the foam cell formation induced by ox-LDL in macrophages. **A** Cytotoxicity of Gastrodin at different concentrations on macrophages. **B** Oil red O staining of the macrophages. **C** Quantification of TC content in macrophages. **D** Effects of Gastrodin on mRNA expression levels of the genes related cholesterol influx and efflux. **E** Protein levels of SRB1 and ABCA1 in macrophages. All data represent the mean ± SEM. ^*^P < 0.05, ^**^P < 0.01, ^***^P < 0.001 versus control group. ^#^P < 0.05, ^##^P < 0.01 versus ox-LDL group

Macrophages were incubated with Gastrodin at different concentrations. MTT assay were used to analyze cell viability. As shown in Fig. 4A, cytotoxicity of gastrodin elevated with the increase of its concentrations. Therefore, 5 μM and 25 μM were chosen as low concentration and high concentration for the subsequent experiments.

### Gastrodin inhibited foam cell formation by promoting cholesterol efflux

Aim to elucidate the potential mechanism by which Gastrodin attenuated atherosclerosis lesion, foam cell formation model by ox-LDL was constructed in macrophages. Lipid accumulation were induced in macrophages incubated with ox-LDL, and then the intracellular total cholesterol levels were tested by Oil red O staining. As shown in Fig. 4B, ox-LDL induced much more foam cell formation than the control group, and the high concentration of Gastrodin treatment significantly decreased the number of foam cells, however, the low concentration of Gastrodin had no obvious effect on foam cell formation. These results are consistent with lipid extraction of macrophages as shown in Fig. 4C.

To elucidate the mechanism underlying the protective effect of Gastrodin on foam cell formation, the mRNA expressions of key genes involved in lipid efflux (SR-B1, ABCA1 and ABCG1) and influx (SRA and CD36) were detected. Our results demonstrated that mRNA expression and protein level of SR-B1 and ABCA1 were obviously up-regulated in Gastrodin treatment group at high concentration of 25 μM. While the mRNA expression and protein level of SRA were significantly down-regulated after Gastrodin treatment, but other genes expression was not significantly affected by Gastrodin. This finding demonstrated that Gastrodin promote cholesterol efflux through upregulating cholesterol efflux genes like SR-B1 and ABCA1. What’s more, Gastrodin inhibited SRA-dependent lipid uptake.

### Gastrodin inhibited LPS induced inflammation in macrophage

**Fig. 5:**
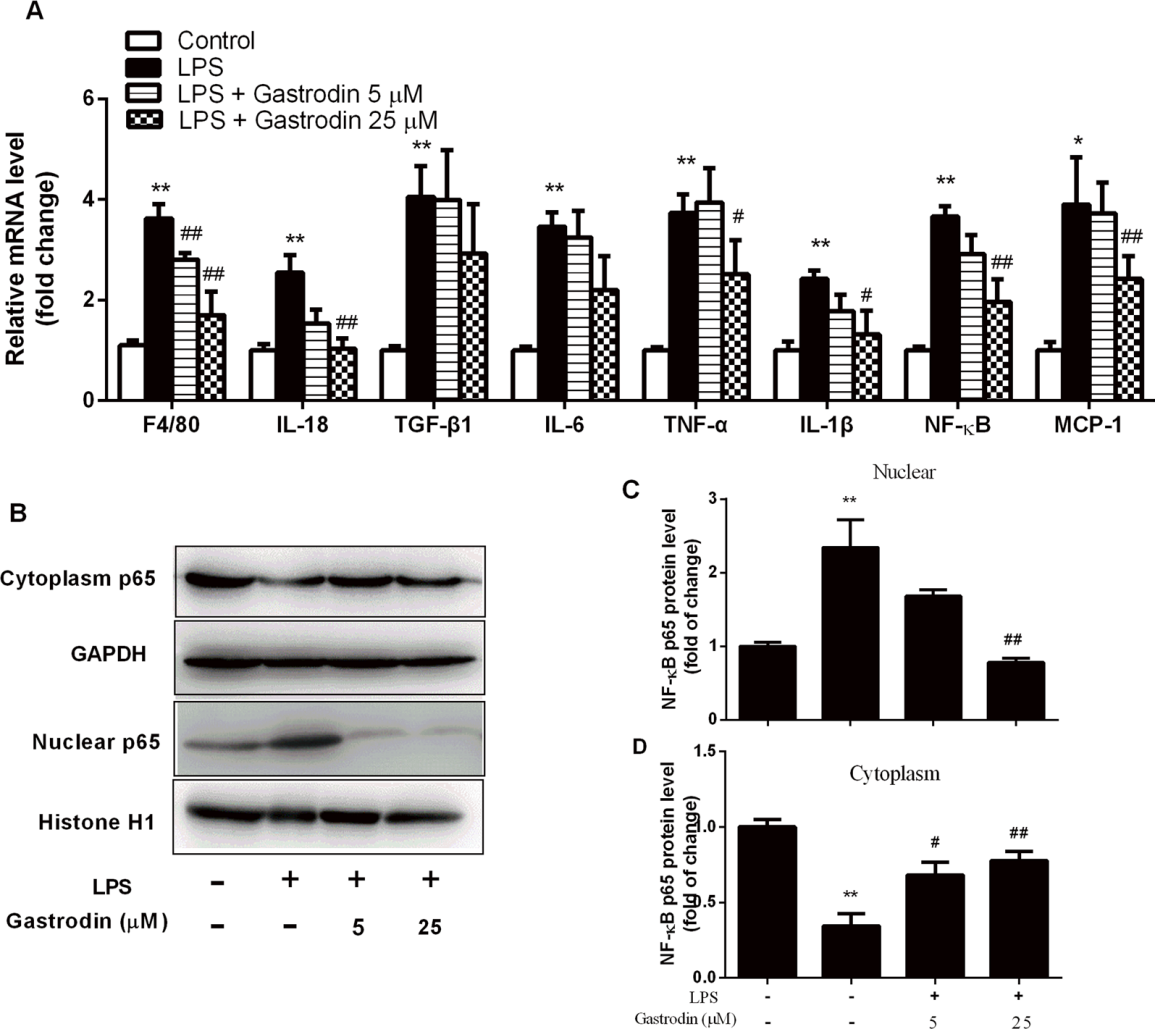
Effects of Gastrodin on mRNA expression of inflammatory factors and protein level of NF-κB p65 in LPS stimulated macrophages. **A** Effect of Gastrodin on mRNA expression of F4/80/ IL-18 /TGF-β1/IL-6/ TNF-α/ IL-1β/NF-Κb/MCP-1. **B**, **C** and **D** Protein level and quantitative analysis of NF-κB p65 in macrophages. Data are presented as mean ± SEM, n = 8, ^*^ P < 0.05, ^**^P < 0.01 versus control group. ^#^P < 0.05, ^##^P < 0.01 versus LPS group

Peritoneal macrophages were treated with 1 ng/ml LPS and 25 μM Gastrodin for 24 h to establish atherosclerosis inflammation cell models. As expected, LPS induced mRNA upregulation of inflammatory cytokines, including F4/80, IL-18, TNF-α, IL-6, TGF-β1, IL-1β, NF-κB and MCP-1 (Fig. 5A). And, high concentration (25 μM) of Gastrodin significantly decreased mRNA expression of F4/80, TNF-α, IL-18, IL-6, IL-1β, NF-κB and MCP-1. We further proved the protein level of TNF-α, NF-κB, IL-18 and IL-1β were significantly upregulated in LPS treated macrophages. Furthermore, Gastrodin supplementation significantly inhibited TNF-α, NF-κB, IL-18 and IL-1β protein levels. Therefore, our findings demonstrated that Gastrodin suppresses LPS induced inflammatory response of macrophage in vitro.

## Discussion

In this study, we validated for the first time the anti-atherosclerotic activity of Gastrodin and the underlying mechanisms. In an in vivo atherosclerosis mouse model, Gastrodin inhibited the inflammation and macrophage accumulation in atherosclerosis lesions. Furthermore, in vitro cell models, we demonstrated that Gastrodin inhibit foam cell formation and LPS-induced atherosclerosis inflammation response in macrophage. Mechanistically, on one hand, Gastrodin treatment represses SRA-mediated lipid influx in macrophages, but augments gene expression related to cholesterol efflux including SRB-1 and ABCA1. On the other hand, Gastrodin inhibits LPS-induced inflammation response through downregulating NF-κB signaling pathway.

Atherosclerosis is a chronic inflammatory disease that is characterized by atherosclerosis plaque formation [19, 20]. Predominant accumulation of cholesterol-laden macrophages and then formation of foam cells are the main drivers of atherosclerosis development [21]. Additionally, macrophages can release inflammatory factors and accelerate atherosclerosis progression [22]. In view of these pathophysiological mechanisms, anti-inflammation or lipid-lowering drugs have been used to alleviate atherosclerosis in clinic. And, it is necessary to find out effective remedies to improve macrophage cholesterol homeostasis and inflammation for atherosclerosis treatment.

Gastrodin is the major active compound extracted from *Gastrodiae elata Blume*, which has been reported to have anti-inflammation and anti-oxidative activity [11, 23], but the effect of Gastrodin on atherosclerosis remains unknown. Gastrodin is widely used to treat neurodegenerative diseases and nervous system diseases in clinic [14, 24], such as Alzheimer’s disease, dizziness, epilepsy and neurasthenia. In addition, recent evidence demonstrates that Gastrodin inhibits foam cell formation through induced lysosomal function and enhanced autophagic activity via AMPK-FoxO1-TFEB signaling axis [18]. However, the above study is only based on in vitro cell model, further in vivo study is needed to validate Gastrodin’s anti-atherosclerosis therapeutic potential.

In the present study, our findings for the first time showed Gastrodin alleviates atherosclerosis lesions and macrophages accumulation in murine model. Macrophage accumulate modified LDL such as oxidized LDL (ox-LDL) and advanced glycation end product LDL (AGE-LDL). Scavenger receptors (SRs) such as SR-A and CD36 are responsible for the uptake of modified LDL leading to foam cell formation and progressing atherosclerotic lesions [25, 26]. In our study, we found that the mRNA expression levels of CD36 and SRA were increased in the foam cells, and high concentration of Gastrodin significantly down-regulated SRA mRNA expression. The efflux process for the removal of cholesterol from macrophages is mediated by reverse cholesterol transporters (RCT). ATP-binding cassette transporter ABCA1, ABCG1 and scavenger receptor BI (SR-BI) are involved in RCT [27]. We found that the mRNA expression and protein level of ABCA1 and SR-BI were increased in the foam cells, but not ABCG1, which were markedly augmented after Gastrodin treatment. Taken together, these results indicated that Gastrodin inhibits SRA-dependent cholesterol influx and promotes SR-BI and ABCA1-depenndent cholesterol efflux.

In addition to impaired lipid homeostasis, inflammation within vessel walls plays a critical role in atherosclerosis. Our data demonstrate that Gastrodin showed markedly anti-inflammatory effects on atherosclerosis progression. Firstly, in vivo experiments, HFD induced significantly release of pro-inflammatory cytokines, such as IL-1β, IL-18 and TNF-α, and Gastrodin decreased the expression levels of aorta NF-κB p65, leading to reduced above inflammatory cytokines levels. NF-κB is closely related to inflammation and atherosclerosis development, and suppression of LPS-induced NF-κB signaling attenuates atherosclerosis development [28, 29]. Secondly, in LPS-induced inflammation macrophage cells, Gastrodin significantly inhibited NF-κB gene expression, and regulate nuclear NF-κB translocation. Our findings indicate that anti-inflammatory effects of Gastrodin were mainly through down-regulating NF-κB pathways. Previous study have reported that Gastrodin attenuated IL-1β-induced inflammation and suppressed apoptosis by inhibiting NF-κB pathways in rat chondrocytes [30]. And our results further demonstrated that Gastrodin inhibit LPS-induced nuclear translocation of NF-κB. Altogether, Gastrodin ameliorate inflammatory response in atherosclerosis progression through inhibiting NF-κB signaling pathway.

## Limitations

First, our results showed that Gastrodin treatment decrease the nuclear NF-κB expression, suggesting that the NF-κB pathway may be related to the underlying anti-inflammatory mechanism of Gastrodin. However, further researches are needed to explain the specific mechanism of the regulation of inflammation in macrophages. Moreover, this is only an animal experiment, and it must be proven in clinical trials before it can be used in humans.

## Conclusion

In conclusion, our results provide direct evidence, for the first time, that Gastrodin exhibited significant anti-atherosclerotic activity by inhibition of macrophage-derived foam cell formation and inflammatory response. Our study is the first to validate Gastrodin’s anti-atherosclerosis effect in vivo and in vitro data. Thus, this study provides theoretical basis for the clinical potential of Gastrodin on atherosclerosis treatment.

## Data Availability

The datasets generated during and/or analysed during the current study are available from the corresponding author on reasonable request.

## Abbreviations

Ldlr: Low-density lipoprotein receptor
HFD: High fat diet
ox-LDL: Oxidized low-density lipoprotein
LPS: Lipopolysaccharide
SRB-I: Scavenger receptor class B
ABCA1: ATP-binding cassette transporter A1
CVD: Cardiovascular disease
IGF2: Insulin-like growth factor type 2 gene
NAFLD: Nonalcoholic fatty liver disease
TG: Triglyceride
TC: Total cholesterol
SRA: Scavenger receptors class A
SR: Scavenger receptor
AGE-LDL: Advanced glycation end product LDL
RCT: Reverse cholesterol transporters

## References

[1] Bjorkegren JLM, Lusis AJ (2022). Atherosclerosis: recent developments. Cell.

[2] Kong P, Cui ZY, Huang XF, Zhang DD, Guo RJ, Han M (2022). Inflammation and atherosclerosis: signaling pathways and therapeutic intervention. Signal Transduct Target Ther.

[3] Negre-Salvayre A, Guerby P, Gayral S, Laffargue M, Salvayre R (2020). Role of reactive oxygen species in atherosclerosis: lessons from murine genetic models. Free Radic Biol Med.

[4] Kriszbacher I, Koppan M, Bodis J (2005). Inflammation, atherosclerosis, and coronary artery disease. N Engl J Med.

[5] Hansson GK (2005). Inflammation, atherosclerosis, and coronary artery disease. N Engl J Med.

[6] Xiang Q, Tian F, Xu J, Du X, Zhang S, Liu L (2022). New insight into dyslipidemia-induced cellular senescence in atherosclerosis. Biol Rev Camb Philos Soc.

[7] Susser LI, Rayner KJ (2022). Through the layers: how macrophages drive atherosclerosis across the vessel wall. J Clin Invest.

[8] Koelwyn GJ, Corr EM, Erbay E, Moore KJ (2018). Regulation of macrophage immunometabolism in atherosclerosis. Nat Immunol.

[9] Rohatgi A, Khera A, Berry JD, Givens EG, Ayers CR, Wedin KE, Neeland IJ, Yuhanna IS, Rader DR, de Lemos JA, Shaul PW (2014). HDL cholesterol efflux capacity and incident cardiovascular events. N Engl J Med.

[10] Wolf D, Ley K (2019). Immunity and inflammation in atherosclerosis. Circ Res.

[11] Qu LL, Yu B, Li Z, Jiang WX, Jiang JD, Kong WJ (2016). Gastrodin ameliorates oxidative stress and proinflammatory response in nonalcoholic fatty liver disease through the AMPK/Nrf2 pathway. Phytother Res.

[12] Hsieh CL, Chiang SY, Cheng KS, Lin YH, Tang NY, Lee CJ, Pon CZ, Hsieh CT (2001). Anticonvulsive and free radical scavenging activities of *Gastrodia elata* Bl. in kainic acid-treated rats. Am J Chin Med.

[13] An SJ, Park SK, Hwang IK, Choi SY, Kim SK, Kwon OS, Jung SJ, Baek NI, Lee HY, Won MH, Kang TC (2003). Gastrodin decreases immunoreactivities of gamma-aminobutyric acid shunt enzymes in the hippocampus of seizure-sensitive gerbils. J Neurosci Res.

[14] Liu Y, Gao J, Peng M, Meng H, Ma H, Cai P, Xu Y, Zhao Q, Si G (2018). A review on central nervous system effects of gastrodin. Front Pharmacol.

[15] Lu J, Ma X, Gao WC, Zhang X, Fu Y, Liu Q, Tian L, Qin XD, Yang W, Zheng HY, Zheng CB (2021). Gastrodin exerts cardioprotective action via inhibition of insulin-like growth factor type 2/insulin-like growth factor type 2 receptor expression in cardiac hypertrophy. ACS Omega.

[16] Zhang Q, Yang YM, Yu GY (2008). Effects of gastrodin injection on blood pressure and vasoactive substances in treatment of old patients with refractory hypertension: a randomized controlled trial. Zhong Xi Yi Jie He Xue Bao.

[17] Wan J, Zhang Y, Yang D, Liang Y, Yang L, Hu S, Liu Z, Fang Q, Tian S, Ding Y (2021). Gastrodin improves nonalcoholic fatty liver disease through activation of the adenosine monophosphate-activated protein kinase signaling pathway. Hepatology.

[18] Tao J, Yang P, Xie L, Pu Y, Guo J, Jiao J, Sun L, Lu D (2021). Gastrodin induces lysosomal biogenesis and autophagy to prevent the formation of foam cells via AMPK-FoxO1-TFEB signalling axis. J Cell Mol Med.

[19] Hansson GK, Hermansson A (2011). The immune system in atherosclerosis. Nat Immunol.

[20] Libby P, Ridker PM, Hansson GK, Leducq Transatlantic Network on A (2009). Inflammation in atherosclerosis: from pathophysiology to practice. J Am Coll Cardiol.

[21] Mallat Z (2014). Macrophages. Arterioscler Thromb Vasc Biol.

[22] Li K, Ching D, Luk FS, Raffai RL (2015). Apolipoprotein E enhances microRNA-146a in monocytes and macrophages to suppress nuclear factor-kappaB-driven inflammation and atherosclerosis. Circ Res.

[23] Yang P, Han Y, Gui L, Sun J, Chen YL, Song R, Guo JZ, Xie YN, Lu D, Sun L (2013). Gastrodin attenuation of the inflammatory response in H9c2 cardiomyocytes involves inhibition of NF-kappaB and MAPKs activation via the phosphatidylinositol 3-kinase signaling. Biochem Pharmacol.

[24] Hu Y, Li C, Shen W (2014). Gastrodin alleviates memory deficits and reduces neuropathology in a mouse model of Alzheimer's disease. Neuropathology.

[25] Rahaman SO, Lennon DJ, Febbraio M, Podrez EA, Hazen SL, Silverstein RL (2006). A CD36-dependent signaling cascade is necessary for macrophage foam cell formation. Cell Metab.

[26] Linton MF, Fazio S (2001). Class A scavenger receptors, macrophages, and atherosclerosis. Curr Opin Lipidol.

[27] Moore KJ, Sheedy FJ, Fisher EA (2013). Macrophages in atherosclerosis: a dynamic balance. Nat Rev Immunol.

[28] Cuaz-Perolin C, Billiet L, Bauge E, Copin C, Scott-Algara D, Genze F, Buchele B, Syrovets T, Simmet T, Rouis M (2008). Antiinflammatory and antiatherogenic effects of the NF-kappaB inhibitor acetyl-11-keto-beta-boswellic acid in LPS-challenged ApoE-/- mice. Arterioscler Thromb Vasc Biol.

[29] Han J, Chen D, Liu D, Zhu Y (2018). Modafinil attenuates inflammation via inhibiting Akt/NF-kappaB pathway in apoE-deficient mouse model of atherosclerosis. Inflammopharmacology.

[30] Chen J, Gu YT, Xie JJ, Wu CC, Xuan J, Guo WJ, Yan YZ, Chen L, Wu YS, Zhang XL (2018). Gastrodin reduces IL-1beta-induced apoptosis, inflammation, and matrix catabolism in osteoarthritis chondrocytes and attenuates rat cartilage degeneration in vivo. Biomed Pharmacother.

